# Improvement of pneumatosis cystoides intestinalis after steroid tapering in a patient with bronchial asthma: a case report

**DOI:** 10.1186/1752-1947-7-163

**Published:** 2013-06-26

**Authors:** Akiko Ezuka, Kenichi Kawana, Hajime Nagase, Hirokazu Takahashi, Atsushi Nakajima

**Affiliations:** 1 Gastroenterology Division, Yokohama Rosai Hospital, Yokohama, Japan; 2 Gastroenterology Division, Yokohama City University Graduate School of Medicine, 3-9 Fuku-ura Kanazawaku, Yokohama, 236-0004, Japan

**Keywords:** Asthma, Pneumatosis cystoides intestinalis, Prednisolone

## Abstract

**Introduction:**

We report the case of a patient who was diagnosed as having pneumatosis cystoides intestinalis while being treated with prednisolone for bronchial asthma. Even before we had experienced a case of this, the relationship between pneumatosis cystoides intestinalis and prednisolone was unclear. In this case, pneumatosis cystoides intestinalis was improved with the reduction of prednisolone, and therefore we thought a direct relationship between pneumatosis cystoides intestinalis and prednisolone might become clear, such as whether it is dose dependent.

**Case presentation:**

A 62-year-old Japanese woman had been treated for bronchial asthma for approximately 40 years. She presented with abdominal distension, and a radiographic examination showed intraperitoneal free gas and intramural gas, suggestive of pneumatosis cystoides intestinalis. However, when her prednisolone dose was decreased from 30mg to 0mg for approximately a year because of improvement in her asthma symptoms, her abdominal symptom resolved, and the frequency of her bowel movements returned to normal.

**Conclusion:**

Amelioration of pneumatosis cystoides intestinalis was observed with tapering of the prednisolone, suggesting that prednisolone may have been involved in the pathogenesis of pneumatosis cystoides intestinalis in this patient.

## Introduction

Pneumatosis cystoides intestinalis (PCI) is a rare condition in which multiple pneumatocysts develop in the submucosa or subserosa of the colon. PCI was first reported in anatomic dissection by DuVernoi in 1730, and Meyer was the first to use the term in 1925 [1,2]. PCI is characterized by multiple gas-filled cysts in the wall of the large intestine [1], and is an unexpected radiologic finding in many cases [1]. Abdominal pain is the most frequent complaint. We encountered a case of PCI apparently induced by a steroid used for asthma treatment, which resolved with tapering of the steroid.

## Case presentation

**Figure 1:**
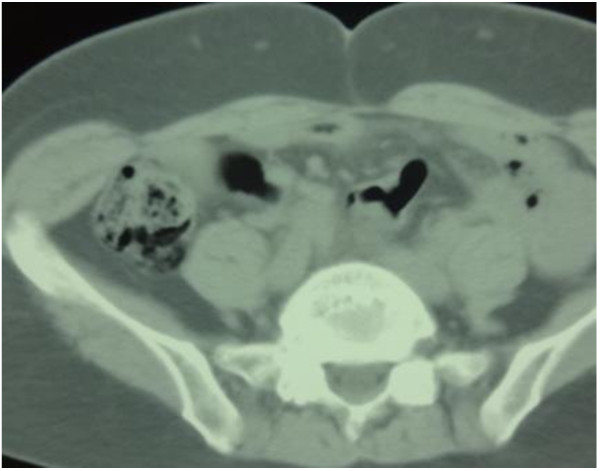
Abdominal computed tomography scan examination showing extensive appearance of intramural gas in the colon.

**Figure 2:**
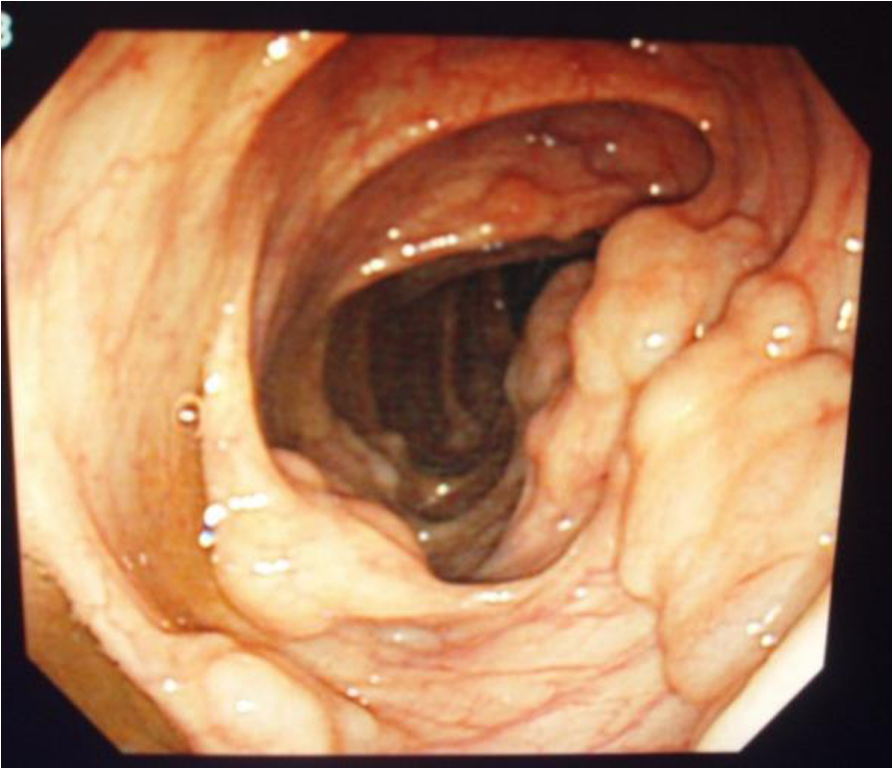
Colonoscopy showing soft polypoid lesions in the digestive tract.

**Figure 3:**
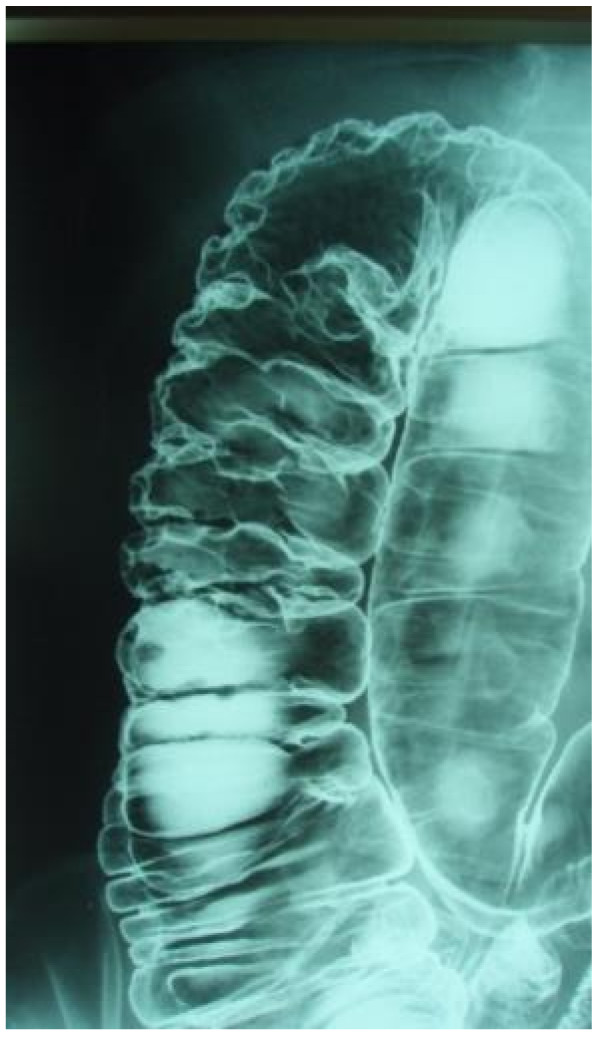
Lower gastrointestinal tract contrast study showing air accumulation in the ascending colon.

**Figure 4:**
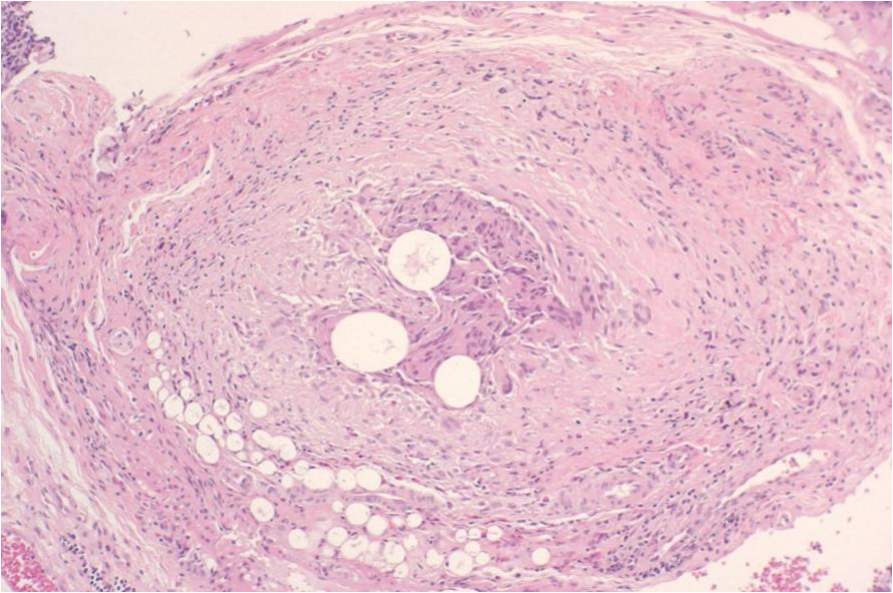
Pathological findings showed pneumatic cysts in the submucosa and subserosa (×50).

A 62-year-old Japanese woman was observed for approximately half a year because of upper abdominal pain, however, an upper gastrointestinal endoscopy, fluoroscopic examination and abdominal computed tomography (CT) revealed no abnormal findings. Thereafter, the patient’s symptom settled. Four years later, she visited our hospital because of a feeling of fullness in the abdomen and increase in the frequency of bowel movements. An abdominal CT revealed extensive appearance of intramural gas in the colon (Figure 1), particularly in the ascending portion. No abnormality was noted on the surface of the intestinal wall by colonoscopy, except for soft polypoid lesions (Figures 2 and 3). The soft polypoid lesions were 6mm in diameter, on average, with a maximum diameter of 33mm. Pathological examination revealed a cluster of pneumatic cysts in the submucosa and subserosa of the colon, based on which the diagnosis of PCI was made (Figure 4). There was no evidence of inflammation, despite her abdominal symptoms and laboratory findings, including elevated serum C-reactive protein levels and leukocytosis. She has had hypertension, hyperlipidemia, and asthma for decades. She was taking the following routine daily medications: amlodipine besylate, *Lactobacillus casei*, albumin tannate, and butropium bromide. These medications were continued during the treatment period for bronchial asthma. Prednisolone (PSL) was started at a dose of 30mg/day. During the observation period, the severity of the bronchial asthma symptoms fluctuated. The PSL dose was gradually tapered as the asthma symptoms improved; PSL 30mg was administered each time the asthmatic symptoms increased in severity during the observation period. PSL was the only drug whose dose was modified during the same period.

## Discussion

PCI is characterized by the development of multiple submucosal or subserosal pneumatocysts in the submucosa or subserosa of the colon [3-5]. An abdominal X-ray and CT demonstrated pneumoperitoneum with massive air accumulation in the intestinal wall, particularly in the colon, leading to the diagnosis of PCI [6]. The etiological mechanisms of PCI are unclear, although PCI has been reported to develop in association with raised intra-abdominal pressure due to ileus surgery, colonoscopy, pulmonary diseases such as chronic bronchitis and emphysema, trichloroethylene exposure, connective tissue disorders, and use of immune-suppressants and alpha-glucosidase inhibitors [3,4]. PSL reduces the number of lymphocytes in the gastrointestinal wall, particularly in the Peyer’s patches, impairing the bowel defense barrier system [7], and causes fragility of the intestinal wall, decreased bowel movements, and excessive intraluminal air production [6]. Both bronchial asthma and PSL can cause PCI. Therefore, we could not exclude either as the cause of the PCI in this patient. However, the patient had received PSL for a prolonged period of time, and the possibility of her developing diabetes was high; however, there were no abnormalities in the blood glucose concentration or serum HbA1c, therefore, she did not receive any antidiabetic drug, particularly voglibose. The suspected etiopathogenic mechanism is alveolar air leakage secondary to high airway pressures [8]. Although the very high intrathoracic pressure observed in our patient may have been responsible for such air leakage and contributed to the development of the PCI, gas collections in most cases of PCI associated with pulmonary obstructive diseases occur in the large bowel. It is a secondary finding associated with a wide variety of underlying gastrointestinal or extragastrointestinal diseases, such as autoimmune disease (scleroderma, dermatomyositis), inflammatory disease (inflammatory bowel disease), and infectious diseases (*Clostridium difficile*, human immunodeficiency virus), pulmonary disease (chronic obstructive pulmonary disease, bronchial asthma), drugs (corticosteroids, immunosuppressive therapy), and trauma (blunt abdominal trauma, endoscopy) [1]. In addition, there are also reports on the relationship of pneumomediastinum with PCI. Both pneumomediastinum and PCI can arise as a result of mesenteric ischemia, and therefore occur in patients with ischemic diseases such as acute mesenteric ischemia [9-12].

## Conclusions

This patient was diagnosed as having asthma when she was 25-years old, and had been under treatment with PSL for 13 years. She suffered from abdominal pain two or three times per month after having been initiated on treatment with PSL 30mg, however, the abdominal symptom resolved after tapering of PSL. Therefore, development of PCI in the present patient was probably related to the use of PSL 30mg. High-dose PSL and the long-term use of the drug have been reported as risk factors for the development of PCI. She showed dramatic improvement following tapering of the steroid, and is asymptomatic at the present time. At first, we considered whether the onset of PCI depended on the amount of PSL, but the relationship remains unclear. However, because the PCI had improved in parallel with the reduction of the steroid dose, we suggest that the gradual decrease of the steroid dose may have contributed significantly to the improvement of the PCI in our patient. It is noted that PSL is one of the drugs most commonly associated with the development of PCI [1,6]. If a patient being treated with PSL complains of abdominal pain, testing for suspected PCI is needed [1].

## Consent

Written informed consent was obtained from the patient for publication of this manuscript and accompanying images. A copy of the written consent is available for review by the Editor-in-Chief of this journal.

## Competing interests

The authors declare that they have no competing interests.

## Authors’ contributions

KK and HN recruited our patient for participation and follow-up at the hospital. AE, HT and AN were major contributors in writing the manuscript. All authors read and approved the final manuscript.
